# Left heart catheterization using the single catheter radial approach with the multipurpose catheter: Teaching an old dog new tricks

**DOI:** 10.1002/clc.23942

**Published:** 2022-11-29

**Authors:** Bryan Kindya, John Lisko, Errol Inci, Jaikirshan Khatri, William Nicholson, Spencer King

**Affiliations:** ^1^ Department of Medicine, Division of Cardiology Emory University School of Medicine Atlanta Georgia USA; ^2^ Department of Medicine, Division of Cardiology Cleveland Clinic Cleveland Ohio USA

**Keywords:** angiography, imaging, multipurpose catheter, radial

## 1 INTRODUCTION

It is an honor to contribute to a tribute to my friend Richard Conti, a true pioneer in Cardiology, and in his earlier years a denizen of the Cath Lab. Dick coached me on transeptal puncture and years later I had the chance to return the favor by helping him get started with stenting. For many years at far flung venues I looked forward to getting together with him in the ACCEL office to talk about late breaking trials and new breakthroughs. From Sylvan Weinberg to Dick to Fred Bove and my brief stint chairing ACCEL, the interactions with Dick were a highlight of the meetings as well as the post meeting activities, and golf. It was all fun and I miss it. It seems appropriate in honoring Dick to pick a Cath Lab topic that is both old and new.

## 2 BACKGROUND

Medical innovation should arise from unmet clinical need. Some who have struggled with the single catheter multipurpose (MP) technique for coronary angiography may think it developed from a diabolical plot to torture cardiology fellows, however the actual motivation was less devious, as at the time there was a significant unmet clinical need. In the 1960s coronary angiography (when performed at all) was mostly done by the Sones technique from a cutdown on the right brachial artery using a single woven dacron catheter. Mastering the maneuvers to intubate the coronary artery ostia was not a trivial task and cardiologists who did learn the technical tricks needed were understandably proud operators and mentors. Radiologists more accustomed to the percutaneous approach were prone to use the technique developed by Melvin Judkins with preformed catheters designed to find the coronary arteries by themselves. These were inserted by the Seldinger method into the femoral artery over a guidewire. Early on the images were recorded on cut film plates using a Franklin changer. The angiograms were not visible in real time but awaited film development. Douglass Adams and Herbert Abrams in Boston noted a serious complication after catheter exchanges over the guidewire without any protective sheath.^1^ Thrombotic and tissue material would be picked up and carried on the exchanged catheter to embolize to the coronary. The idea of using a single catheter from the femoral artery to eliminate exchanges, facilitate left ventriculography, and to enable switching from one coronary artery to the other came from Fred Schoonmaker, my (SK) partner in Denver. He initially tried to use the Sones catheter, but it did not have the torque control needed. We settled on an 8Fr Cordis catheter and after several modifications, with our names on them, we agreed that the stock MP A2 catheter worked best. After I returned to Emory Hospital in 1972, we described the technique and our combined experience in 6800 cases.^2^ Generations of fellows learned the technique from John Douglas and myself, and in so doing gained a great deal of knowledge of the variations in aortic root anatomy and coronary artery origins. Eight french became seven french, and then six. Some abandoned the technique for the easier Judkins catheters but others continued and trained successive generations of cardiologists with the MP catheter.

Now, the femoral approach is infrequently used but the MP catheter survives in some quarters. With advances in both catheter and vascular access technology, radial access has become the preferable access site for cardiac catheterization, with a variety of advantages compared to femoral access, including bleeding, safety, and possibly mortality as well in certain circumstances.^3^, ^4^, ^5^, ^6^, ^7^ I observe with awe the interventional skills being taught to our fellows as well as the diagnostic skills practiced with the MP catheter from the wrist. Some of these fourth‐generation fellows and their mentors will describe what has evolved from this 53‐year‐old concept.

## 3 CORONARY ANATOMY

**Figure 1:**
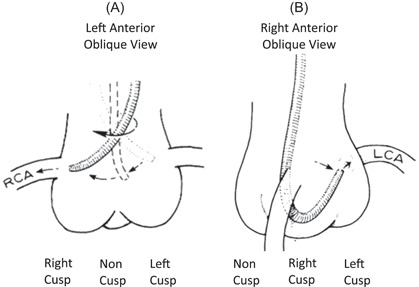
Coronary cusp and coronary artery anatomy from the LAO and RAO positions. (A) Anatomy from the LAO position. (B) Anatomy from the RAO position. LAO, left anterior oblique; RAO, right anterior oblique.

An important first step in understanding engagement of the coronary arteries with the MP catheter is understanding the anatomy of the aortic root and coronary arteries from the left anterior oblique (LAO) and right anterior oblique (RAO) positions. In LAO, the right coronary cusp, and by association the right coronary artery (RCA), originate anteriorly which is the leftmost cusp on the screen from the view of the operator. The left coronary cusp and left coronary arteries arise from the rightmost cusp on the screen, with the non‐coronary cusp in between the two (Figure 1A). In contrast to this, from the RAO position, the noncoronary cusp is the leftmost cusp on the screen for the operator, while the left remains on the right side of the screen, with the right coronary cusp in between the two (Figure 1B).

## 4 RIGHT CORONARY ENGAGEMENT

The RCA originates off the right coronary cusp which is the more anterior of the coronary arteries. Given this, it is best engaged from the LAO position, usually 30° of LAO. In general, there are three different approaches to cannulation of the RCA from the radial approach with the MP catheter.

### 4.1 Direct cannulation

**Figure 2:**
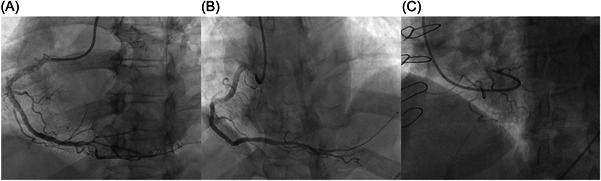
Cannulation of the RCA ostium with the MP catheter from three different approaches, all shown in LAO. (A) Engagement with direct cannulation from above. (B) Cannulation from below via curling the MP in the root in a superior takeoff RCA. (C) “Back door” engagement of the RCA in a nondominant RCA on diagnostic cath. LAO, left anterior oblique; MP, multipurpose; RAO, right anterior oblique; RCA, right coronary artery.

While there are several options from multiple vendors for MP catheters available, we find the Cordis 6 French MP A2 Super Torque to provide the best responsiveness when torquing. The B2 version of the same catheter may be preferable for coronary engagement in women and patients with a small aortic root. From the radial approach, the RCA can commonly be cannulated directly from above without having to form or shape the catheter in the aortic root. From the LAO position, the catheter is torqued to point anteriorly (toward the left side of the screen) and manipulated up and down the aorta with subtle changes in orientation via clockwise and counterclockwise rotation to change the orientation of the tip of the catheter. Once it approaches the RCA ostium, it will cannulate the RCA (Figure 2A). Careful attention must be taken to notice the deflection and change in the tip of the MP catheter as it enters the RCA ostium as if it is continued to be advanced it can dive deep into the mid RCA. Once engaged and confirmation of an appropriate pressure waveform is confirmed, angiography can be performed.

### 4.2 Cannulation from below

Sometimes, direct cannulation is difficult or not achievable. A common situation where this is observed is instances where the takeoff of the RCA is superior in nature making direct engagement from above not possible. In this situation the artery is best engaged from below. To perform this, the catheter is pointed toward the left side of the screen in the LAO position and advanced into the right coronary cusp. The catheter is slowly advanced farther into the cusp and a small loop is formed. Once this small loop is formed, the catheter is gently advanced farther and will jump up to engage the right coronary ostium (Figure 2B).

### 4.3 The “Back Door” approach

When cannulating from below, sometimes it is quite difficult to form the tight loop needed to jump up into the RCA ostium. When this occurs, and the catheter is advanced farther, instead of forming a tight loop the catheter will instead form a larger loop around the base of the right coronary cusp itself. In this situation, the catheter is advanced farther with subtle rotation to advance it into the RCA ostium to perform angiography (Figure 2C). Care must be taken to not push too far, or the catheter will commonly prolapse into the left ventricle, performing a left heart catheterization. Clockwise rotation of the catheter helps prevent this from happening.

### 4.4 The high and anterior takeoff RCA

A commonly encountered anatomic variation making engagement of the RCA difficult is when it originates from a higher and more anterior position than usual. Difficulties in this engagement can be due to recognizing the high and anterior takeoff of the RCA in general, as well as locating and cannulating its ostium. Due to the reach of the MP catheter compared to other preshaped catheters used for RCA cannulation, it can commonly cannulate and perform angiography on high and anterior right coronary arteries from the radial approach as well. The engagement of them is similar to the direct cannulation approach, with the exception that the catheter is pointed more anterior (commonly directly at the screen toward the operator in LAO) and moved up and down much higher than traditional cannulation from above, very similar to how it can be used for vein graft cannulation from a femoral approach. Using this approach, a significant amount of high and anterior right coronary arteries can be visualized with the MP from the radial approach. When this is not successful, other catheters to consider include the 3DRC catheter as well as the AL diagnostic catheter.

## 5 LEFT CORONARY ARTERY ENGAGEMENT

**Figure 3:**
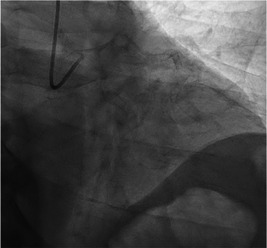
Appearance of the MP after engagement of the left main from the RAO position, achieved by direct engagement of the left coronary cusp from the radial approach. MP, multipurpose; RAO, right anterior oblique.

There are generally two approaches to engaging the left coronary artery with a MP catheter from a radial approach. For both, the fluoroscope should be moved to a RAO position at about 30°. This view separates the noncoronary cusp from the left and right, with the former being more posterior toward the spine. The first approach, and slightly more difficult from a radial approach, is the traditional approach from a femoral position described by Schoonmaker and King.^2^, ^8^, ^9^ The catheter tip is pointed toward the noncoronary cusp and advanced until a loop is created. The catheter is then torqued clockwise which causes an eventual flip to orient the catheter in the left coronary cusp, allowing advancement to engage the left coronary system. In contrast to the femoral approach, the MP catheter can commonly engage the left coronary cusp directly from the radial approach, bypassing the need for flipping the catheter from the noncusp altogether. This leads to the second and more commonly used method to cannulate the left main from the radial approach. The tip is pointed at approximately a 5 O'clock position and advanced directly into the left coronary cusp. From here advancement will cause the tip to slide up the coronary cusp with the catheter in the bottom of the cusp. Continued advancement and counterclockwise rotation will rotate the catheter tip further up the cusp and more posterior until it is properly engaged in the ostium of the left main artery (Figure 3). Once engaged, it is not uncommon that the catheter tip is directed at the roof of the left main which can be relieved by slight withdrawal and sometimes clockwise rotation of the catheter which will lift the belly of the catheter off the left coronary cusp and allow the catheter tip to be more co‐axial with the coronary artery.

For inexperienced operators, it may be difficult to distinguish whether the catheter tip is in the left or right coronary cusp in the RAO projection as these are partially superimposed. Since the MP catheter can easily engage the left coronary cusp directly from the radial approach, it may be useful to start left coronary engagement in the LAO projection. Once the catheter is in the left cusp, switch to RAO to engage the left main. A small test injection in RAO can inform the operator of appropriate adjustments to achieve coaxial engagement with the left main. Specifically, if the catheter tip is screen‐right of the left main it is too far anterior, and counter‐clockwise torque will bring the catheter tip posterior. Conversely, if the catheter tip is screen‐left of the left main it is too far posterior and clockwise torque will bring the catheter anterior. These simple correction in the RAO projection can also facilitate difficult left coronary engagement with non‐MP catheters which are generally manipulated exclusively in the LAO projection.

## 6 LEFT HEART CATHETERIZATION

**Figure 4:**
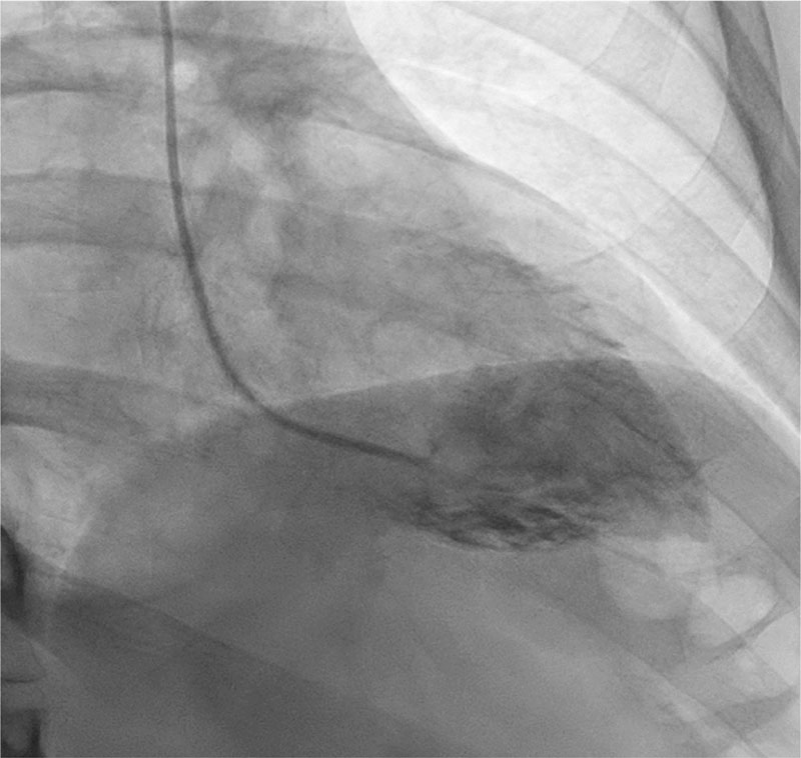
An RAO projection of the MP catheter seated in the left ventricle along the ventricular axis used to perform a left ventriculogram. MP, multipurpose; RAO, right anterior oblique.

The MP catheter is well suited for advancement into the left ventricle to obtain left ventricular end diastolic pressure, and given its proximal side port it is quite safe to use to perform a ventriculogram. Frequently, the catheter will naturally fall into the ventricle when attempting to engage the left or RCA. If this occurs, the catheter can be advanced into the ventricle and positioned parallel to the long axis of the heart, which is best seen in the RAO view (Figure 4). Crossing the aortic valve is performed by first retracting the MP into the sinotubular junction. In a RAO projection, the catheter tip is positioned toward the spine (the noncoronary cusp) and advanced until a large loop is formed. This loop will often cross into the ventricle with gentle advancement. If it does not, the loop is rotated clockwise until the MP is viewed in an en‐face projection and then advanced through the aortic valve. Ventricular ectopy can occur and typically resolves with slight clockwise or counter‐clockwise torque. In the RAO projection, clockwise torque will direct the catheter tip down and counter‐clockwise torque will direct the catheter tip up. A small injection confirms the catheter position before a hand injection or power‐injection to perform a ventriculogram.

## 7 CONCLUSION

Left heart catheterization and coronary angiography remains the gold standard for assessment of coronary artery disease. While the original description of performing a left heart catheterization with the MP catheter was described from the femoral approach, the MP is also viable as a single catheter technique to perform left heart catheterization and coronary angiography from the radial approach as well. Due to its design as a shapeable catheter with a side hole proximal to the distal port, its versatility is notable and an entire left heart catheterization with coronary angiography can be performed with a single catheter from the radial approach. In experienced hands, this catheter represents a safe, efficient, and practical tool that has stood the test of time in the cardiac catheterization lab.

## References

[1] Adams DF , Fraser DB , Abrams HL . The complications of coronary arteriography. Circulation. 1973;48:609‐618. 4726245 10.1161/01.cir.48.3.609

[2] Schoonmaker FW , King SB . Coronary arteriography by the single catheter percutaneous femoral technique: experience in 6,800 cases. Circulation. 1974;50:735‐740. 4547535 10.1161/01.cir.50.4.735

[3] Kiemeneij F , Laarman GJ , de Melker E . Transradial artery coronary angioplasty. Am Heart J. 1995;129(1):1‐7. 7817902 10.1016/0002-8703(95)90034-9

[4] Mann JT , Cubeddu MG , Schneider JE , Arrowood M . Right radial access for ptca: a prospective study demonstrates reduced complications and hospital charges. J Invasive Cardiol. 1996;8(Suppl D):40D‐44D. 10785786

[5] Campeau L . Percutaneous radial artery approach for coronary angiography. Cathet Cardiovasc Diagn. 1989;16(1):3‐7. 2912567 10.1002/ccd.1810160103

[6] Jolly SS , Yusuf S , Cairns J , et al. Radial versus femoral access for coronary angiography and intervention in patients with acute coronary syndromes (rival): a randomised, parallel group, multicentre trial. The Lancet. 2011;377(9775):1409‐1420. 10.1016/S0140-6736(11)60404-2 21470671

[7] Romagnoli E , Biondi‐Zoccai G , Sciahbasi A , et al. Radial versus femoral randomized investigation in ST‐Segment elevation acute coronary syndrome. J Am Coll Cardiol. 2012;60(24):2481‐2489. 22858390 10.1016/j.jacc.2012.06.017

[8] Kacharava AG , Clements SD , Zafari AM . Pocket Guide to Diagnostic Cardiac Catheterization. Cardiotext Publishing; 2016:104‐109.

[9] King SB , Douglas JS . Coronary arteriography and angioplasty. McGraw‐Hill; 1984.

